# Protecting Health from Climate Change

**DOI:** 10.4103/0970-0218.42042

**Published:** 2008-07

**Authors:** JS Thakur

**Affiliations:** School of Public Health, Department of Community Medicine, Post Graduate Institute of Medical Education and Research (PGIMER), Chandigarh - 160 012, India. E-mail: jsthakur_in@yahoo.co.in

Climate change is one of the most important global environmental challenges facing humanity with implications for food production, natural ecosystem, freshwater supply, health, etc. During the last 100 years, human activities related to the burning of fossil fuels, deforestation, and agriculture have led to a 35% increase in the carbon dioxide (CO_2_) levels in the atmosphere, causing increased trapping of heat and warming of the earth's atmosphere. The Fourth Assessment Report (AR-4) of the Intergovernmental Panel on Climate Change (IPCC) states that most of the observed increase in the global temperatures since the mid-20th century was very likely due to the increase in concentration of anthropogenic greenhouse gases (GHGs).(1) Of the six CHGs, CO_2_ accounted for 63%, methane 24%, nitrous oxide 10%, and other gases the remaining 3% of the carbon equivalent emissions in 2000.(2) High accuracy measurements of atmospheric CO_2_ concentration initiated by Charles Davis Keeling in 1958, constituted the master time series with formal international coordination of meteorological observation from ships commenced in 1853. The IPCC also reports that the global average sea level rose at an average rate of 1.8 mm/year from 1961 to 2003. The total rise in the sea level during the 20th century was estimated to be 0.17 m. The global surface warming projections will vary between 1.1 and 6.4°C and mean sea level is projected to rise by 30-60 cm by the year 2100.

India is a large developing country with nearly 700 million rural population directly depending on climate-sensitive sectors (agriculture, forests, and fisheries) and natural resources (such as water, biodiversity, mangroves, coastal zones and grasslands) for their subsistence and livelihoods. The contribution of India to the cumulative global CO_2_ emissions from 1980 to 2003 is only 3.11%. Thus, historically and at present, India's share in the carbon stock in the atmosphere is relatively very small when compared with the population. India's carbon emissions per person are 20th of those of the US and a 10th of most Western Europe and Japan. Climate change is likely to impact all the natural ecosystems as well as socioeconomic systems as shown by the National Communications Report of India to the UN Framework Convention on Climate Change (UNFCCC).(2)

The latest high-resolution climate change scenarios and projections for India, based on Regional Climate Modeling (RCM) system, known as PRECIS developed by Hadley Center and applied for India using IPCC scenarios A2 and B2 shows an annual mean surface temperature rise by the end of century, ranging from 3 to 5°C under A2 scenario and 2.5 to 4°C under B2 scenario, with warming more pronounced in the northern parts of India.(2) A 20% rise in all India summer monsoon rainfall and further rise in rainfall is projected over all states except Punjab, Rajasthan, and Tamil Nadu, which show a slight decrease. Extremes in maximum and minimum temperatures are also expected to increase and similarly extreme precipitation also shows substantial increases, particularly over the west coast of India and west central India. Glaciers in Himalaya are melting and may lead to glacier lake outburst floods as occurred in Himachal Pradesh. As glaciers are the source of drinking and irrigation water in mountains and Indo-Gangetic region in India, the long-term reduction in annual snowmelt is expected to result in water insecurity and more interstate conflicts in these region.(3) Most of the states in North, including Punjab, Haryana, Rajasthan, Uttar Pradesh, Madhya Pradesh, and North East, are dependent upon river water with origin in Himalayas. Urban cities in developing countries, including India, will be more affected due to fast pace of development, industrialization and increasing vehicular traffic.(4)

These climatic changes will cause disruption of the ecosystem's services to support human health and livelihood, and will impact health systems.(5) The IPCC projects an increase in malnutrition and consequent disorders, with implications for child growth and development. The disruption in rainfall patterns can be expected to lead to an increased burden of diarrhoeal disease and vector-borne diseases. WHO estimates that the modest climate change that has occurred since 1970, claims 150,000 lives annually. WHO has rightly chosen this issue as theme of World Health Day, 2008. The current and emerging climate change-related health risks in Asia, including India, include heat stress, water, and foodborne diseases (e.g., cholera and other diarrhoeal diseases) associated with extreme weather events (e.g., heat waves, storms, floods and flash floods, and droughts); vector-borne diseases (e.g., dengue and malaria); respiratory diseases due to air pollution; airborne allergens, food, and water security issues; malnutrition; and psychosocial concerns from displacement.(5) These risks and diseases are not new, and the health sector is already tackling these problems. However, the capacity to cope with potentially increasing levels of these risks and diseases is limited, particularly in developing countries.

Since early 1990s, international efforts have created the climate change regime, the center piece of which is the UNFCCC and its instruments, the Kyoto Protocol, and Marrakesh Accords, which details rules for its implementations. These currently existing multilateral instruments themselves are not adequate to meet the twin challenges of mitigation and adaptation. They do, however, provide a basis for development of the multilateral regimes. The three Kyoto mechanisms are joint implementation (JI), clean development mechanism (CDM), and emission trading (ET).(2) Only CDM is relevant to developing countries such as India. Developing countries could view CDM as an opportunity not only to attract capital investment and environmentally sustainable technologies (ESTs), but also to implement innovative technical, institutional, and financial interventions to promote energy efficiency, renewable energy, and forestry activities that contribute to sustainable development. Most national governments have signed and ratified the Kyoto Protocol, aimed at reducing GHG emissions, but its first commitment period ends in 2012. Regional framework devised at Bali could also be the guiding force.(6) There is an urgent need to incorporate health concerns into the decisions and actions of different sector-wise approaches to mitigate and adapt to climate change.

India has completed four nationally coordinated assessments of climate change projections, impacts, and mitigation. Recently, National Action Plan on Climate Change (NAPCC) has also been released with many good initiatives including a proposal for a National Solar Mission. Key Ministries of Government of India, such as Environment and Forest, Water Resources and Health and that of states, should devise urgent policy initiatives and implementation of NAPCC to mitigate the effect of climate change in country. Green technology, projects, and industries must be encouraged and promoted by instituting awards and others must be taxed. It must be supplemented by individual efforts leading to collective efforts for saving energy. There is need to promote the use of non-motorized transport systems (e.g., bicycles), mass rapid transport system, and fewer private vehicles by taxing more than one or two vehicles per family for reducing GHG emissions, improving air quality, and making more people physically active. Such an approach would also help in reducing the emerging epidemic of non-communicable diseases including road-traffic accidents. Public health associations in India such as Indian Public Health Association and Indian Association of Preventive and Social Medicine must take lead by organizing thematic seminar and special sessions in conferences. Thus, India has a significant stake in scientific advancement as well as an international understanding to promote mitigation and adaptation. This requires improved scientific understanding, capacity building, networking, and broad consultation processes. Let us join hands to save planet earth and secure our survival and of future generations.

## References

[1] Intergovernmental Panel on Climate Change (IPCC) Report of working group II. Climate change impacts, Adaptations and Vulnerability.

[2] Sathaye J, Shukla PR, Ravindranath NH (2006). Climate change, sustainable development and India: Global and national concerns. Curr Sci.

[3] (2005). Human Health Impacts from Climate Variability and Climate Change in the Hindu Kush-Himalaya Region: A Report of an Interregional Workshop, India.

[4] Lendrum DC, Corvalan C, Neira M (2007). Climate change and developing country cities: Implications for environmental health and equity. J Urban Health.

[5] Lendrum DC, Corvalan C, Neira M (2007). Global climate change: Implications for international public health policy. Bull WHO.

[6] (2007). Regional Framework for action to protect human health from effect of climate change in the South East Asia and Pacific region.

